# The influence of bearing surfaces on revisions due to dislocations in total hip arthroplasty

**DOI:** 10.1007/s10856-021-06598-4

**Published:** 2021-09-15

**Authors:** Francesco Castagnini, Barbara Bordini, Monica Cosentino, Cristina Ancarani, Federica Mariotti, Federico Biondi, Cesare Faldini, Francesco Traina

**Affiliations:** 1 Ortopedia-Traumatologia e Chirurgia protesica e dei reimpianti d’anca e di ginocchio, IRCCS Istituto Ortopedico Rizzoli, Via Pupilli 1, 40136 Bologna, Italy; 2 Laboratorio di Tecnologia Medica, IRCCS Istituto Ortopedico Rizzoli, Via di Barbiano 1/10, 40136 Bologna, Italy; 3 Head of Clinica Ortopedica e Traumatologica I, IRCCS Istituto Ortopedico Rizzoli, Via Pupilli 1, 40136 Bologna, Italy; 4 Orthopaedics and Traumatology, University of Bologna DIBINEM, 40123 Bologna, Italy; 5 Head of Ortopedia-Traumatologia e Chirurgia protesica e dei reimpianti d’anca e di ginocchio, IRCCS Istituto Ortopedico Rizzoli, Via Pupilli 1, 40136 Bologna, Italy

## Abstract

**Introduction:**

Recurrent dislocations are still the most frequent reason for revision in total hip arthroplasty (THA). The impact of bearing surfaces on dislocations is still controversial. We hypothesized that: (1) bearing surfaces influence the revisions due to dislocations; (2) ceramic-on-ceramic reduced the revisions for dislocations in adjusted models; (3) Delta-on-Delta bearings reduced the revisions for dislocations in comparison to surfaces with cross-linked polyethylene.

**Materials and methods:**

The regional arthroplasty registry was enquired about bearing surfaces and revisions for dislocations and instability. Unadjusted and adjusted rates were provided, including sex, age (<65 years or ≥65 years), head diameter (≤28 mm or >28 mm; <36 mm or ≥36 mm) as variables. 44,065 THAs were included.

**Results:**

The rate of revisions for dislocations was significantly lower in ceramic-on-ceramic and metal-on-metal bearings (unadjusted rates). After adjusting for age, sex, and head size (36 and 28 mm), hard-on-hard bearings were protective (*p* < 0.05): ceramic-on-ceramic had a lower risk of revisions due to dislocation than ceramic-on-polyethylene (HR 1.6, 95% CI 1.2–2.2 *p* = 0.0009). The rate of revisions for dislocation was similar in bearings with cross-linked polyethylene and Delta-on-Delta articulations, in unadjusted and adjusted models.

**Conclusion:**

Bearings with conventional polyethylene were more predisposed to dislocations. Currently adopted bearings exerted no significant influence on revisions due to dislocations. These findings could be primarily related to wear, but due to the time distribution, soft tissue envelopes and surface tension may also play a role. Pre-clinical biomechanical evaluations and prospective matched cohort studies are required to draw definitive conclusions.

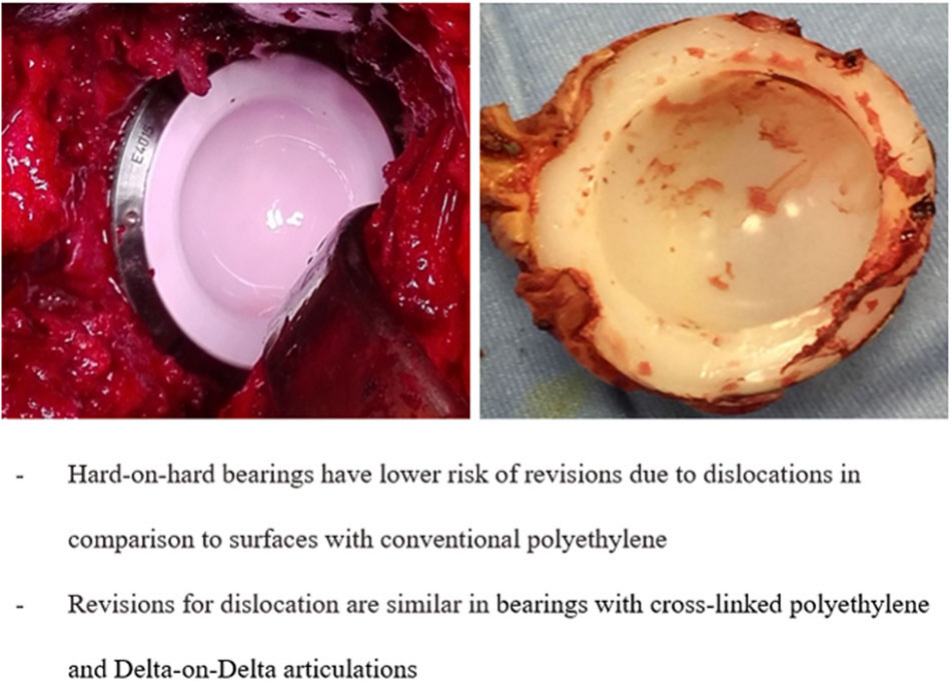

## Introduction

Dislocations are still the most frequent reason for revision in total hip arthroplasty (THA) and the incidence is steadily rising [1, 2]. Head size, patient’s age, surgeon experience, surgical approach and implant type have been advocated as the major predictive factors influencing THA dislocations [3–5]. The role of bearing surfaces in preventing dislocations has been discussed, with controversial conclusions. While Hernigou et al. reported that ceramic couplings were less susceptible to dislocations, Sexton et al. noted that the revision rates due to dislocations were statistically higher in primary ceramic-on-ceramic THAs [3, 6, 7]. The two most recent registry studies by Pitto et al. and Shah et al. concluded that bearing surfaces have a little, non-significant impact on revisions for dislocations [2, 8].

Thus, the current literature about bearing surfaces and revisions for dislocations is contradictive and only partially examines the most recent bearing surfaces, Delta ceramics and cross-linked polyethylene (XLPE) [8]. Hence, a large registry population of primary THAs was investigated to assess whether: (1) bearing surfaces impacted the revision rates due to dislocations (unadjusted rates); (2) ceramic-on-ceramic reduced the revisions for dislocations after model adjustment for gender, age (<65 years or ≥65 years), head diameter (≤28 or >28 mm; <36 or ≥36 mm); (3) Delta-on-Delta bearings reduced the revision rates due to dislocations in comparison to modern articulations as metal-on-XLPE and Delta-on-XLPE in unadjusted and adjusted populations.

## Materials and methods

The Emilia Romagna region registry RIPO includes primary and revision hip, knee and shoulder arthroplasties, performed in 68 Orthopedic Units since January 2000 [5, 9]. The demographics of patients, the diagnoses leading to THA, the features of surgical procedures, the type (batch and code) and fixation of implants are collected similarly to the most important national registries. A specific paper form including all the data is filled in by the surgeon and then it is sent to RIPO. Crossover comparisons with other databases and missing data retrievals are routinely performed to improve registry accuracy. The report includes only resident patients. This method avoids the bias due to the lack of follow-up data: surgical revisions on resident patients, even when performed elsewhere in Italy, are always billed back to the reference region and captured by the registry. RIPO achieved a capture rate of 98% [5, 9].

RIPO was enquired about the correlation between bearing surfaces and rates of revisions for primary instability/dislocations in primary cementless THAs. The inclusion criteria of the present study were all the primary cementless THAs performed for primary osteoarthritis and avascular necrosis, from January 2000 to December 2016.

The exclusion criteria were:

-THAs performed for diagnoses other than primary osteoarthritis or avascular necrosis
-cemented or hybrid implants
-dual mobility articulations
-hip resurfacing procedures
-metal-on-metal THAs with heads larger than 36 mm

The exclusion criteria aimed to exclude patients with higher risks of dislocation (e.g., dysplastic) and all the implants adopted in high-risk patients (e.g., dual mobility) or correlated with soft tissue pathology (metal-on-metal implants with large heads possibly causing metallosis) [3, 10].

Demographics and implant features were collected and compared. The end-points of the study were revisions for dislocations (multiple dislocation occurring after three months) and revisions for primary instability (revisions due to subluxation/impingement related events occurring in the first three months) [5].

First, an unadjusted evaluation of bearings and revisions due to dislocations was performed. Then, bearings were compared using a regression model to assess the effects of independent predictive factors for dislocations (inferred from literature) on revisions due to dislocations/primary instability: sex, age, head size [2, 3]. Lastly, the most widespread and recent articulations, Delta-on-Delta (Biolox Delta, Ceramtec, Plochingen, Germany), Delta-on-XLPE, metal-on-XLPE, were compared. Oxinium-on-XLPE (Smith and Nephew, Andover, US) bearings were not evaluated due to the low number of implants involved.

44,065 THAs met the inclusion criteria and were analyzed (Table 1). Ceramic-on-ceramic (COC) surfaces were implanted in the vast majority of THAs (53%). Ceramic-on-polyethylene (COP) articulations accounted for the 26.4% of the whole implants, whereas metal-on-metal (MOM) (0.4%) and metal-on-polyethylene (MOP) (17.1%) were occasionally adopted. 293 (0.7%) cases were revised due to recurrent dislocations or primary instability. THAs were stratified by bearing surfaces: demographics and implant features were detailed in Table 1. Similarly, the demographics and implant features of the most recent articulations were specified in Table 2.

**Table 1:** Demographics and implant features were similar in the four groups

Demographics and implant features				
	COC	COP	MOM	MOP
Number of implants	23,348	11,630	1560	7527
Mean age (years)	66.6	71.5	63.9	71.9
Female sex (%)	55%	58%	55%	60%
BMI (kg/m^2^) between 19–25	32%	34%	35%	33%
Weight <80 kg (%)	63%	67%	65%	66%
Head size <36 mm (%)	44%	73%	100%	92%
Head size ≤28 mm (%)	14%	47%	85%	78%
Lateral approach (%)	48%	61%	80%	69%
Posterolateral approach (%)	32%	33%	19%	25%
Revisions for dislocation	110	100	8	75
Follow-up (years)	5.6	6.1	10.1	8.4

**Table 2:** Demographic and implant features of the most recent articulations (Delta-on-Delta, Delta-on-XLPE, metal-on-XLPE) showed inhomogeneous distribution between the three cohorts

Demographics and implant features			
	Delta-on-Delta	Delta-on-XLPE	Metal-on-XLPE
Number of implants	16,672	4648	3179
Mean age (years)	67	72.6	73
Female sex (%)	54%	56%	60%
BMI (kg/m^2^) between 19–25	32%	33%	34%
Weight <80 kg (%)	63%	63%	66%
Head size <36 mm (%)	32%	51%	81%
Head size ≤28 mm (%)	1%	13%	66%
Lateral approach (%)	44%	55%	71%
Posterolateral approach (%)	33%	36%	21%
Revisions for dislocation	66	27	21
Follow-up (years)	3.9	3	7

Institutional board review was waived due to the nature of registry studies, collecting anonymous data as a standard practice.

## Statistical analysis

Patient demographics, implant features and reasons for revision were analyzed using descriptive statistics, such as means, ranges, and percentages. Values were compared using a *t*-test or Chi-square test. Kaplan–Meier survival analysis was performed. Survival times of unrevised implants were calculated considering the last date of observation or the date of death. The log-rank test was used to compare survivorships of the cohorts. Survival data were analyzed using Cox multiple regression model: Wald test was used to calculate the *p* values. The proportionality hazards assumption (HR) was tested by the Schoenfeld residual method; age, gender and head sizes used for adjustment fulfilled the proportional hazard assumption for the whole period. The threshold for significance was *p* = 0.05 for all the tests. All statistical analyses were performed using JMP®, Version <x>. SAS Institute Inc., Cary, NC, 1989–2007.

## Results

**Fig. 1:**
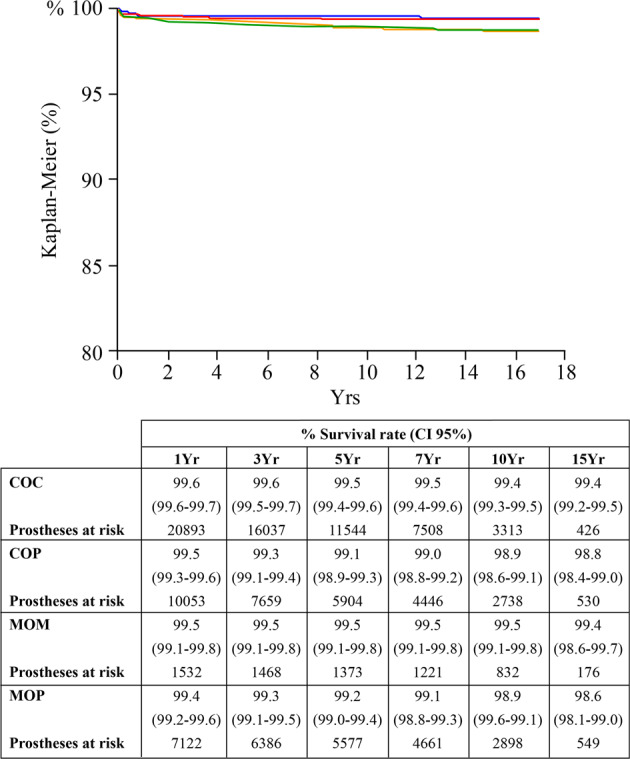
The Kaplan–Meier curves (endpoint: revisions due to recurrent dislocations and primary instability) showed that COC implants were significantly less prone to dislocations than COP THAs (unadjusted rates). COC: red line; COP: green line; MOM: blue line; MOP: orange line

The risk of revision for dislocations was significantly lower for hard-on-hard bearings (COC and MOM) than for hard-on-soft articulations (COP and MOP) (unadjusted rates; *p* < 0.05) (Fig. 1).

The adjusted risk ratios for gender, age (<65 years or ≥65 years), head diameter (≤28 mm or >28 mm; <36 mm or ≥36 mm) were calculated using the Cox multiple regression model.

The regression model adjusted for age gender and head diameter (28 mm) showed that bearing surfaces statistically influenced the revisions due to dislocations (*p* = 0.03) (Table 3). COP couplings were more at risk of dislocations than COC (HR 1.4, 95% CI 1.0–1.9, *p* = 0.04) and MOM (HR 2.4, 95% CI 1.2–5.4, *p* = 0.008). MOP articulations were more prone to dislocations than MOM (HR 2.1, 95% CI 1.1–4.8, *p* = 0.03). No differences were detected between MOM and COC couplings (HR 0.6, 95% CI 0.3–1.1, *p* = 0.1).

**Table 3:** Cox regression model investigating the influence of bearings on revisions for dislocation was adjusted for age, gender, and head diameter (28 mm): in bold, significant findings

Bearings		HR	CI 95%	*p*
COC	MOM	1.74	0.88–3.95	0.1148
**COC**	**COP**	**0.73**	**0.54**–**0.98**	**0.0384**
COC	MOP	0.82	0.58–1.16	0.2637
MOM	COC	0.57	0.25–1.13	0.1148
**MOM**	**COP**	**0.41**	**0.19**–**0.81**	**0.0082**
**MOM**	**MOP**	**0.47**	**0.21**–**0.92**	**0.0273**
**COP**	**COC**	**1.37**	**1.01**–**1.85**	**0.0384**
**COP**	**MOM**	**2.39**	**1.23**–**5.37**	**0.0082**
COP	MOP	1.13	0.83–1.53	0.4354
MOP	COC	1.22	0.86–1.71	0.2637
**MOP**	**MOM**	**2.12**	**1.08**–**4.79**	**0.0273**
MOP	COP	0.88	0.65–1.20	0.4354

When the model was adjusted for age, gender, and head diameter (36 mm), once again bearing surfaces influenced the rate of revisions for dislocations (*p* = 0.001) (Table 4). There was a higher risk of revisions due to dislocations in COP cohort than in COC implants (HR 1.6, 95% CI 1.2–2.2 *p* = 0.0009) and in MOM THAs (HR 2.2, 95% CI 1.1–4.9, *p* = 0.02). No differences were detected between MOM and COC articulations (HR 0.7, 95% CI 0.3–1.4, *p* = 0.4).

**Table 4:** Cox regression model investigating the influence of bearings on revisions for dislocation was adjusted for age, gender, and head diameter (36 mm): in bold, significant findings

Bearings		HR	CI 95%	*p*
COC	MOM	1.35	0.69–3.03	0.4070
**COC**	**COP**	**0.61**	**0.46**–**0.82**	**0.0009**
**COC**	**MOP**	**0.63**	**0.46**–**0.87**	**0.0049**
MOM	COC	0.74	0.33–1.45	0.4070
**MOM**	**COP**	**0.45**	**0.20**–**0.89**	**0.0186**
MOM	MOP	0.47	0.21–0.92	0.0249
**COP**	**COC**	**1.63**	**1.22**–**2.16**	**0.0009**
**COP**	**MOM**	**2.19**	**1.13**–**4.92**	**0.0186**
COP	MOP	1.02	0.76–1.39	0.8833
**MOP**	**COC**	**1.59**	**1.15**–**2.19**	**0.0049**
**MOP**	**MOM**	**2.14**	**1.09**–**4.84**	**0.0249**
MOP	COP	0.98	0.72–1.32	0.8833

**Fig. 2:**
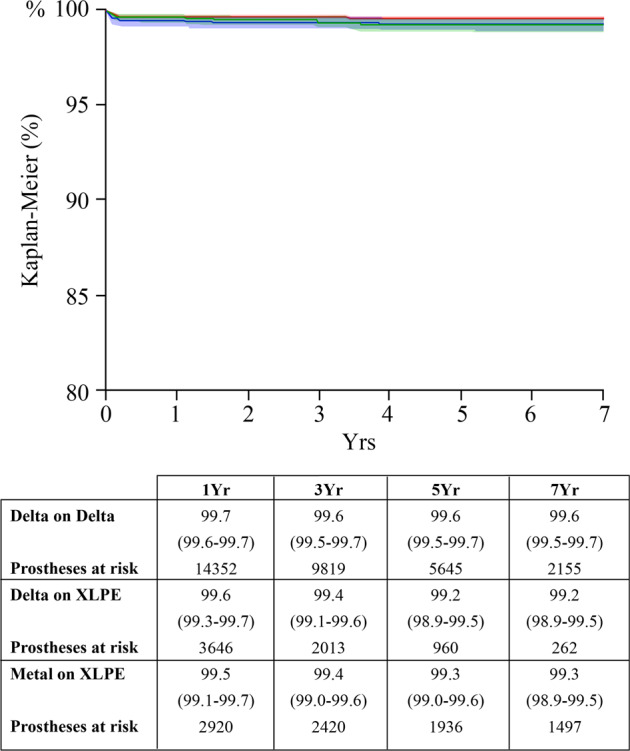
Recent couplings did not show significant differences in terms of revision due to dislocation (Kaplan–Meier curves, endpoint: revisions due to recurrent dislocations and primary instability). Delta-on-Delta: red line. Delta-on-XLPE: green line. Metal-on-XLPE: blue line

When the most recent articulations were analyzed (metal-on-XLPE, Delta-on-XLPE, Delta-on-Delta), the survival rates (endpoint: revisions due to recurrent dislocations/primary instability) were similar in the three cohorts (unadjusted, *p* = 0.06) (Fig. 2). When the three cohorts were stratified for the 28 mm head diameter, no significant differences between couplings were detected in terms of revisions due to dislocations (*p* > 0.05). The articular surfaces did not influence the rate of revisions for dislocations when the model was adjusted for the age, gender, and 36 mm head diameter (*p* = 0.177).

## Discussion

Hard-on-hard (COC and MOM) bearing surfaces exerted a protective influence against revisions due to dislocations and instability when conventional polyethylene was evaluated (Fig. 1). The lower rate of dislocations with hard-on-hard articular surfaces was evident even when some variables related to unstable THA (age, gender, and head size) were controlled (Tables 3 and 4). However, the recent bearings (metal-on-XLPE, Delta-on-XLPE, and Delta-on-Delta) showed no differences in terms of revisions due to dislocations and instability, in unadjusted and adjusted populations (Fig. 2).

The available literature about the impact of bearings on dislocations is controversial: many Authors found out weak correlations [2, 3, 8]. The present report seems to support the findings of Pitto et al., who noticed a weak prevalence of revisions for dislocations in the COP cohort [2]. But, at the same time, the present report was in line with Shah et al. [8]. The Authors evaluated COC and articulations with XLPE: no significant differences in terms of revisions for dislocations were evident apart from metal-on-XLPE bearings with 36 mm heads exhibiting more revisions [8]. The most logical explanation of the predisposition of conventional polyethylene to dislocations in comparison to hard-on-hard bearings and modern articulations with XLPE is obviously wear [6, 7, 11, 12]. However, the wear of conventional polyethylene bearings may easily justify late dislocations occurring at long-terms, but in this report, conventional polyethylene liners showed higher rates of dislocation even at mid-terms (Fig. 1). Thus, advocating only for wear is arduous.

So, other hypotheses explaining the different impact of bearings on dislocations should be made. The first hypothesis concerns the soft tissue envelopes. A thick neo-capsule of clean, dense, regular fibrous tissue full of Type-1 collagen was demonstrated around COC surfaces [13, 14]. Thus, this new capsular reinforcement may effectively stabilize the hip implant even at short-to-midterms, but it was demonstrated only for COC implants [14].

A more comprehensive hypothesis concerns the surface tension [15–18]. As hard-on-hard bearings are highly wettable and smooth materials, the thin fluid film between head and liner generates restraining forces avoiding hip separation during the swing phase of the gait [17, 18]. As a matter of fact, an in vitro evaluation by Clarke et al. demonstrated notable retaining forces for MOM implants at all speeds, more than 12 times higher than COP couplings [16]. These forces translated into the much lower dislocation rates in MOM THAs than in COP implants with the same head size [16]. Komistek et al. ascertained that MOM implants did not exhibit a femoral head separation during the swing phase, differently from MOP articulations [17]. Although literature about ceramics and the cohesiveness of lubricating film is scarce, it is very likely that COC bearings may exhibit a similar behavior, as the smoothness and the wettability of such bearings are even more pronounced than in MOM [18]. It is hard to state whether superficial tension may be advocated also for XLPE, as polymers are known for the low wettability. However, the dependable effects of XLPE articulations against dislocations were observed in other registry studies: biomechanical assessments would be helpful to explain such findings [4, 8].

The main limits of this report are related to the nature of registry studies [2, 3, 5]. Registries allow to detect only revision procedures: thus, conservatively treated dislocations were not captured. The multifactorial etiology of dislocation cannot be properly analyzed by a registry study [2, 3, 12, 13]. Thus, some key factors as the clinical conditions of the patients, surgeon experience, impingement, component malposition and even some specific features of the implants were not assessed. In particular, a proper analysis about elevated liners was not conducted: such devices may reduce dislocation rates, even if this finding is still controversial [19]. On the other side, dislocation is quite a rare event. So, registry studies on large numbers are adequate to allow for proper comparisons, stratify groups and control independent risk factors [13].

## Conclusion

Bearing surfaces with conventional polyethylene were more predisposed to revisions for dislocations and instability. However, with modern bearings with XLPE, no influence of bearing surfaces on revisions due to dislocation is evident. While the most logical explanation of these findings would be wear, the time distribution of the dislocations advocates other causes, like soft tissue envelopes, lubricant conditions and surface tension, all possibly contributing to the better stability of the hard-on-hard and XLPE surfaces. More biomechanical studies about the fluid-film cohesion of the most recent materials and prospective matched cohort studies about the influence of articular surfaces on dislocations may implement the current knowledge about bearings and dislocations.

## Supplementary information

Supplementary Information
