# Supplementary material for: The influence of bearing surfaces on revisions due to dislocations in total hip arthroplasty
Source: J Mater Sci Mater Med. 2021 Sep 15;32(9):123. doi: 10.1007/s10856-021-06598-4 (PMC8443492; doi:10.1007/s10856-021-06598-4)
Supplement: Supplementary file 1 — Supplementary Information [file 10856_2021_6598_MOESM1_ESM.doc]

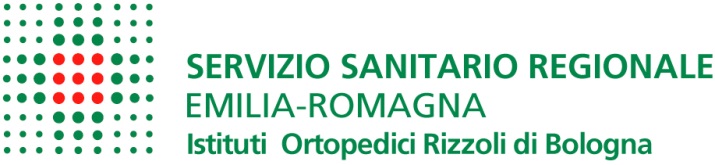


**Registro Implantologia Protesica Ortopedica**

Via di Barbiano, 1/10 – 40136 Bologna, Italy - tel. +39-051-6366880

[https://ripo.cineca.it](https://ripo.cineca.it/)

Bologna, October 17, 2018

**Title: LE SUPERFICI DI CARICO INFLUENZANO IL TASSO DI REVISIONE PER LUSSAZIONE ED INSTABILITÀ**

**Population under study**

The analyses in this report are referred to all primary implant (THA) in the period **01/01/2005 - 31/12/2016**. Failures were recorded up to 31/12/2016.

The extraction from the database was made on 15/10/2018.

Inclusion criteria:

- cementless prostheses, with diagnosis of coxarthritis or necrosis

Exclusion criteria:

- double mobility cups

- large head (>=36mm) metal-on-metal bearing surface procedures

**Analisi preliminare:**

| **Statistiche descrittive** | **Accoppiamento articolare (testina-inserto)** | | | |
| --- | --- | --- | --- | --- |
|  | **cer-cer** | **Cer-pol** | **Met-met** | **Met-pol** |
| ***N° of protesi*** | 23348 | 11630 | 1560 | 7527 |
| *Età media (anni)* | 66,6 | 71,5 | 63,9 | 71,9 |
| ***Follow-up medio*** | 5,6 | 6,1 | 10,1 | 8,4 |
| *Sesso*  Femmine (%) | 55% | 58% | 55% | 60% |
| *Tipo di collo*  Modulare (%) | 14,9% | 9,1% | 0,0% | 0,3% |
| *Fissazione della protesi*  Non cementata (%) | 100 | 100 | 100 | 100 |
| ***% di casi con testina <36*** | 44% | 73% | 100% | 92% |
| ***N° fallimenti per lussazione protesi o instabilità primaria*** | 110 | 100 | 8 | 75 |
| ***% di casi con testina <=28*** | 14% | 47% | 85% | 78% |
| ***% pazienti normopeso*** | 32,4% | 33,5% | 35,2% | 33,0% |
| ***% pazienti con peso inferiore a 80 kg*** | 62,9% | 66,5% | 65,1% | 65,7% |
| ***% di casi con incisione laterale*** | 47,9% | 61,0% | 79,5% | 68,5% |
| ***% di casi con incisione postero laterale*** | 32,2% | 32,6% | 19,0% | 24,6% |

**In dettaglio la distribuzione della dimensione della testina per accoppiamento articolare**

| **DIMENSIONE TESTINA** | **Accoppiamento articolare (testina-inserto)** | | | |
| --- | --- | --- | --- | --- |
|  | **cer-cer** | **Cer-pol** | **Met-met** | **Met-pol** |
| 22 |  |  |  | 16 |
| 26 |  |  |  | 1 |
| 28 | 3264 | 5463 | 1322 | 5837 |
| 32 | 7079 | 3031 | 238 | 1028 |
| 36 | 10654 | 3020 |  | 632 |
| 40 | 2136 | 107 |  | 2 |
| 44 | 131 | 1 |  |  |
| 48 | 78 |  |  |  |

|  | **% survival (Confidence interval 95%)** | | | | | |
| --- | --- | --- | --- | --- | --- | --- |
|  | **1Yr** | **3Yrs** | **5Yrs** | **7Yrs** | **10 Yrs** | **15 Yrs** |
| cer-cer | 99.6  (99.6-99.7) | 99.6  (99.5-99.7) | 99.5  (99.4-99.6) | 99.5  (99.4-99.6) | 99.4  (99.3-99.5) | 99.4  (99.2-99.5) |
| *Prostheses at risk* | 20893 | 16037 | 11544 | 7508 | 3313 | 426 |
| cer-pol | 99.5  (99.3-99.6) | 99.3  (99.1-99.4) | 99.1  (98.9-99.3) | 99.0  (98.8-99.2) | 98.9  (98.6-99.1) | 98.8  (98.4-99.0) |
| *Prostheses at risk* | 10053 | 7659 | 5904 | 4446 | 2738 | 530 |
| met-met | 99.5  (99.1-99.8) | 99.5  (99.1-99.8) | 99.5  (99.1-99.8) | 99.5  (99.1-99.8) | 99.5  (99.1-99.8) | 99.4  (98.6-99.7) |
| *Prostheses at risk* | 1532 | 1468 | 1373 | 1221 | 832 | 176 |
| met-pol | 99.4  (99.2-99.6) | 99.3  (99.1-99.5) | 99.2  (99.0-99.4) | 99.1  (98.8-99.3) | 98.9  (98.6-99.1) | 98.6  (98.1-99.0) |
| *Prostheses at risk* | 7122 | 6386 | 5577 | 4661 | 2898 | 549 |

| **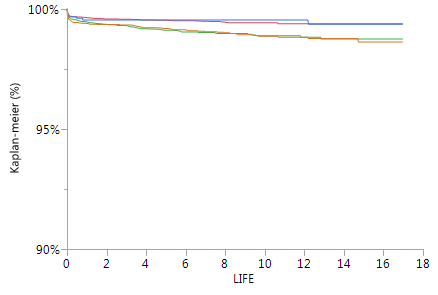** | |  | cer-cer | | --- | --- | |  | cer-pol | |  | met-met | |  | met-pol | |  |  |   La differenza osservata è statisticamente significativa |
| --- | --- | --- | --- | --- | --- | --- | --- | --- | --- | --- | --- |

Infine per analizzare gli ipotetici fattori di rischio si è implementato un modello di regressione di Cox. Le variabili inserite nel modello sono: sesso, età, dimensione della testina, accoppiamento articolare. I risultati sono presentati nella tabella seguente. Risultati sovrapponibili se consideriamo l’età come variabile continua, qui abbiamo preso età <65 vs >=65

**In questa analisi non abbiamo inserito fra i fattori confondenti il collo perché è variabile dipendente con le altre, le met-pol e le met-met non hanno colli modulari**

| **Origine** | **N. param** | **DF** | **Chi-quadrato di Wald** | **Prob>ChiQu** |  |
| --- | --- | --- | --- | --- | --- |
| sesso | 1 | 1 | 3,61024887 | 0,0574 |  |
| classe | 1 | 1 | 4,74935234 | 0,0293* |  |
| DACCOPP | 3 | 3 | 15,731434 | 0,0013* |  |
| classi età | 1 | 1 | 0,10948108 | 0,7407 |  |

| **Livello1** | **/Livello2** | **Rapporto Rischio** | **Prob>ChiQu** | **Inferiore al 95%** | **Superiore al 95%** |
| --- | --- | --- | --- | --- | --- |
| cer-pol | cer-cer | 1,6274033 | 0,0009* | 1,2210547 | 2,1677847 |
| met-met | cer-cer | 0,7430671 | 0,4070 | 0,3297212 | 1,4491418 |
| met-met | cer-pol | 0,4565968 | 0,0186* | 0,2031474 | 0,8866097 |
| met-pol | cer-cer | 1,5910321 | 0,0049* | 1,1527691 | 2,1895336 |
| met-pol | cer-pol | 0,9776508 | 0,8833 | 0,7209814 | 1,3202575 |
| met-pol | met-met | 2,1411688 | 0,0249* | 1,0925962 | 4,8398951 |
| cer-cer | cer-pol | 0,6144758 | 0,0009* | 0,4613004 | 0,8189642 |
| cer-cer | met-met | 1,3457735 | 0,4070 | 0,6900636 | 3,032865 |
| cer-pol | met-met | 2,1901162 | 0,0186* | 1,127892 | 4,922535 |
| cer-cer | met-pol | 0,6285228 | 0,0049* | 0,4567183 | 0,8674764 |
| cer-pol | met-pol | 1,0228601 | 0,8833 | 0,757428 | 1,3869983 |
| met-met | met-pol | 0,4670346 | 0,0249* | 0,206616 | 0,9152512 |


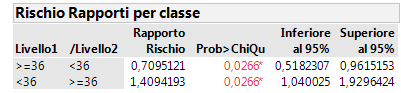


Accoppiamento articolare e dimensione della testina presenta significatività statistica (p<,0001), ti faccio notare che la dimensione della testina in queste classi è significativa ma siamo al limite (lo vedi dall’intervallo di confidenza che quasi tocca il valore 1)

**Questi dati si interpretano in questo modo: vedi stringa evidenziata in giallo…..avere un accoppiamento articolare cer-pol ha un rischio di revisione per lussazione o per instabilità 1.6 volte superiore rispetto ad un accoppiamento articolare cer-cer; questo risultato è valido a parità delle altre variabili inserite nel modello**

Se ripetiamo la stessa analisi inserendo come discriminante la testina del 28 invece che del 36 abbiamo questi risultati del tutto sovrapponibili ai precedenti


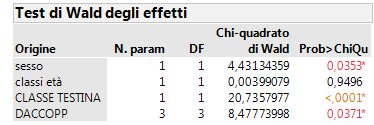


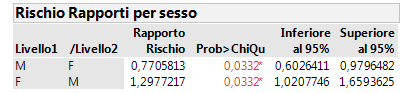


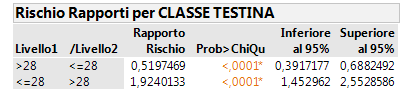


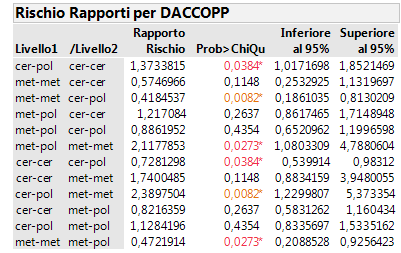


Se vogliamo considerare l’incisione chirurgica e prendendo solo laterale codificata come 2 vs postero laterale codificata come 4 purtroppo perdiamo molti casi, il modello però regge e qui trovi i risultati

| **Incisione chirurgica** | **cer-cer** | **cer-pol** | **met-met** | **met-pol** | **Totale complessivo** |
| --- | --- | --- | --- | --- | --- |
| Laterale | 11127 | 7052 | 1231 | 5127 | 24537 |
| Posterolaterale | 7487 | 3772 | 295 | 1843 | 13397 |
| **Totale complessivo** | **18614** | **10824** | **1526** | **6970** | **37934** |

In questa analisi perde forza il confronto met-met vs cer-pol ma emerge che l’incisione postero laterale ha una probabilità di fallire 3.3 volte maggiore della laterale

| **Origine** | **N. param** | **DF** | **Chi-quadrato di Wald** | **Prob>ChiQu** |  |
| --- | --- | --- | --- | --- | --- |
| sesso | 1 | 1 | 1,85315513 | 0,1734 |  |
| classi età | 1 | 1 | 0,97813148 | 0,3227 |  |
| DACCOPP | 3 | 3 | 11,0684897 | 0,0114* |  |
| classe | 1 | 1 | 9,36286731 | 0,0022* |  |
| incision | 1 | 1 | 86,6625368 | <,0001* |  |


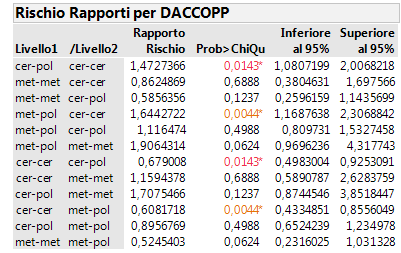


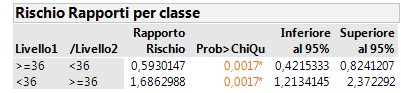


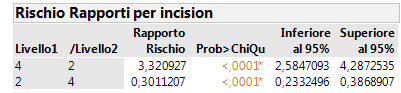


- *i nuovi accoppiamenti, delta-delta, delta XLPE e metallo-XLPE. di questi, farei un'elaborazione anche con grafico di tutti i dati indipendentemente dalla testina, e una con le testine maggiori del 28*

| **Statistiche descrittive** | **Accoppiamento articolare (testina-inserto)** | | |
| --- | --- | --- | --- |
|  | **Delta-delta** | **Delta-xlpe** | **Metallo-xlpe** |
| ***N° of protesi*** | 16672 | 4648 | 3179 |
| *Età media (anni)* | 67,0 | 72,6 | 73,0 |
| ***Follow-up medio*** | 3,9 | 3,0 | 7,0 |
| *Sesso*  Femmine (%) | 53,7 | 56,0 | 60,3 |
| *Tipo di collo*  Modulare (%) | 49,1 | 21,7 | 11,8 |
| *Fissazione della protesi*  Non cementata (%) | 100 | 100 | 100 |
| ***% di casi con testina <36*** | 31,8 | 50,6 | 80,7 |
| ***N° fallimenti per lussazione protesi o instabilità primaria*** | 66 | 27 | 21 |
| ***% di casi con testina <=28*** | 1% | 13% | 66% |
| ***% pazienti normopeso**** | 32,3 | 33,0 | 33,7 |
| ***% pazienti con peso inferiore a 80 kg*** | 62,7 | 63,1 | 65,6 |
| ***% incisioni laterali*** | 44,0% | 55,1% | 70,7% |
| ***% incisioni postero laterali*** | 32,6% | 36,0% | 20,8% |

* purtroppo sono moltissimi i casi con BMi mancanti

In dettaglio la distribuzione della dimensione della testina per accoppiamento articolare

| **DIMENSIONE TESTINA** | **Accoppiamento articolare (testina-inserto)** | | |
| --- | --- | --- | --- |
|  | **Delta-delta** | **Delta-xlpe** | **Metallo-xlpe** |
| 22 |  |  | 6 |
| 26 | 149 | 625 | 2078 |
| 28 | 5149 | 1728 | 481 |
| 32 | 9132 | 2211 | 612 |
| 36 | 2136 | 83 | 2 |
| 40 | 82 | 1 |  |
| 44 | 24 |  |  |
| 48 |  |  | 6 |

| 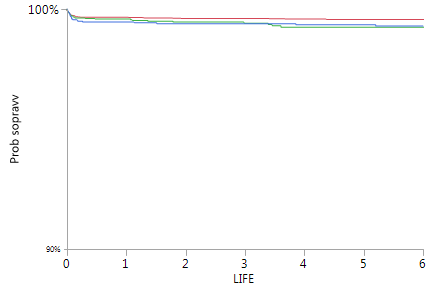 | |  | delta-delta | | --- | --- | |  | delta-XLPE | |  | Metallo-XLPE |   Differenza osservata non statisticamente dignificativa p=0.06 (long-rank test)  Ripetendo questa analisi solo sulle testine maggiori di 28 non cambia il risultato |
| --- | --- | --- | --- | --- | --- | --- | --- |

|  |  | | | |
| --- | --- | --- | --- | --- |
|  | **1Yr** | **3Yrs** | **5Yrs** | **7yrs** |
| **Delta-delta** | 99.7  (99.6-99.7) | 99.6  (99.5-99.7) | 99.6  (99.5-99.7) | 99.6  (99.5-99.7) |
| *Prostheses at risk* | 14352 | 9819 | 5645 | 2155 |
| **Delta-xlpe** | 99.6  (99.3-99.7) | 99.4  (99.1-99.6) | 99.2  (98.9-99.5) | 99.2  (98.9-99.5) |
| *Prostheses at risk* | 3646 | 2013 | 960 | 262 |
| **Metallo-xlpe** | 99.5  (99.1-99.7) | 99.4  (99.0-99.6) | 99.3  (99.0-99.6) | 99.3  (98.9-99.5) |
| *Prostheses at risk* | 2920 | 2420 | 1936 | 1497 |

Per fortuna il BMI e peso sono distribuiti in modo abbastanza sovrapponibile nei gruppi di accoppiamento articolare quindi è una variabile che possiamo considerare non confondente nell’analisi;

Per un trafiletto di statistica andrei su qualcosa del tipo:

Patient demographics, duration of hospitalization, mortality rates, and reasons for revision were analyzed using descriptive statistics, such as means, ranges, and percentages. Where appropriate, values were compared using a t-test or Chi square test (α=0.05). Kaplan-Meier survivorship analysis was performed using revision of any component as the endpoint and survival times of unrevised TKAs taken as the last date of observation (December 31, 2016 or date of death). The log-rank test was used to compare survivorship between the two groups. The Cox multiple regression model for analyzing survival data was considered. The proportionality hazards assumption was tested by the Schoenfeld residual method; age and gender used for adjustment fulfilled the proportional hazard assumption for the all period.

The Wald test was used to calculate the *p* values for data obtained from the Cox multiple regression analyses. Differences between groups were considered statistically significant if the p values were less than 0.05. All statistical analyses were performed using JMP®, Version *<x>*. SAS Institute Inc., Cary, NC, 1989-2007.

poi possiamo aggiornare e adattare in base alle analisi che alla fine decideremo di mettere nel lavoro.

cerco di rispondere puntualmente alle tue domande:

-Le metallo metallo si lussano meno delle cer-poli e delle met-pol in maniera significativa? 
si esatto però attenzione che le met-met sono state da subito depurate dei casi con testa grande, non vorrei che questo creasse confusione nell'interpretazione dei risultati

-Rischio rapporti per classe con testina del 36: le testine >=36 si lussano meno?
esatto e in modo significativo, inserite nel modello con età, sesso e accoppiamento articolare; quindi 

**avere una testina <36 ha un rischio di revisione per lussazione o per instabilità 1.4 volte superiore rispetto ad una testina >=36; questo risultato è valido a parità delle altre variabili inserite nel modello**

-Usando come base la testina del 28 (e non il 36), si può dire lo stesso?

Abbiamo aggiunto questa analisi nel testo ed effettivamente si conferma il risultato
**avere una testina <=28 ha un rischio di revisione per lussazione o per instabilità 1.9 volte superiore rispetto ad una testina >28; questo risultato è valido a parità delle altre variabili inserite nel modello**
